# Lactic acidosis and severe septic shock in metformin users: a cohort study

**DOI:** 10.1186/s13054-015-1180-6

**Published:** 2016-01-15

**Authors:** Keren Doenyas-Barak, Ilia Beberashvili, Ronit Marcus, Shai Efrati

**Affiliations:** 1 Department of Nephrology, Assaf Harofeh Medical Center, affiliated to the Sackler Faculty of Medicine, Tel Aviv University, Zerifin, 70300 Israel; 2 Research and Development Unit, Assaf Harofeh Medical Center, affiliated to the Sackler Faculty of Medicine, Tel Aviv University, Zerifin, 70300 Israel; 3 Internal Medicine C Department, Assaf Harofeh Medical Center, affiliated to the Sackler Faculty of Medicine, Tel Aviv University, Zerifin, 70300 Israel; 4 Sagol School of Neuroscience, Tel Aviv University, Tel Aviv, 69978 Israel

**Keywords:** Lactic acidosis, Metformin, Septic shock, Acute kidney injury

## Abstract

**Background:**

High serum lactate is associated with increased mortality in septic shock patients. Metformin alters lactate metabolism, and may affect its prognostic value. We compared, between metformin users and nonusers, the prognosis of extremely elevated plasma lactate levels in patients with septic shock.

**Methods:**

The electronic medical records (EMR) of patients admitted to the emergency room between January 2011 and June 2013 were reviewed. The study cohort comprised patients with an initial diagnosis of septic shock and blood lactate higher than 10 mmol/L. The selected population was divided into two groups: metformin users (exposed) and metformin nonusers (unexposed). The primary outcome measured was inhospital mortality.

**Results:**

The study included 44 metformin users and 118 nonusers. Metformin users were similar to nonusers with respect to levels of lactate, HCO_3_, and blood pH; however, they were older and had higher incidence rates of cardiovascular disease and acute kidney injury at admission, compared to nonusers. Inhospital mortality rates were significantly lower in the metformin-treated group, 56.8 % vs. 88.1 %, *p* <0.0001.

**Conclusions:**

Though high lactate concentration indicates poor prognosis in septic patients, mortality rate was found to be significantly lower in those who were treated with metformin. This finding may help clinicians in deciding treatment for these patients, who could otherwise be considered too ill for real treatment benefit.

## Background

Lactic acidosis, defined as plasma lactate concentration greater than 5 mM and blood pH lower than 7.35, is common among emergency room (ER) and intensive care unit (ICU) patients. Lactic acidosis generally results from marked tissue hypoperfusion in shock or follows cardiopulmonary arrest; and as such, serves as an indicator of increased mortality risk [1, 2]. Elevated serum lactate is associated with increased mortality, independent of organ failure and shock [3–5]. Metformin, which is currently considered the first choice for oral treatment of type 2 diabetes [6], may interfere with lactate metabolism. However, the prognosis among metformin users, compared to nonusers, of severe sepsis, with highly elevated lactate, is unclear.

The causes of lactic acidosis can be divided into those associated with obviously impaired tissue oxygenation (type A) and those in which systemic impairment in oxygenation does not exist or is not readily apparent (type B). The accumulation of lactate is usually due to enhanced pyruvate production; reduced pyruvate conversion to carbon dioxide and water, or to glucose; and an altered redox state within the cell, in which the pyruvate/lactate ratio shifts toward lactate. Lactic acidosis induced by metformin is typical, but a rare form of type B lactic acidosis, termed metformin-induced lactic acidosis (MILA). When lactic acidosis is diagnosed in a patient treated with metformin, but metformin overdose is not detected, the term metformin-associated lactic acidosis (MALA) better describes the condition. The exact cellular mechanism responsible for lactate accumulation under metformin treatment was unclear for many years, but a recent trial revealed that metformin selectively inhibits the mitochondrial isoform of glycerophosphate dehydrogenase, an enzyme that catalyzes the conversion of glycerophosphate to dihydroxyacetone phosphate (DHAP). As a result, cytosolic DHAP is reduced and the cytosolic NAD–NADH ratio is increased [7]. Cellular accumulation of NAD inhibits the conversion of lactate to pyruvate (limits the use of lactate as gluconeogenic precursors) and culminates in increased plasma concentration of lactate [7]. Metformin also interacts in a dose-dependent manner with hepatic and extrahepatic mitochondrial enzymes [8], and this inhibits global oxygen consumption [9].

The incidence of lactic acidosis in metformin users appears to be very low [10–13]. Nevertheless, MALA remains a concern because of the high case-fatality rate, about 50 %. Most cases of MALA occur in patients with conditions that predispose to hypoperfusion and hypoxemia, such as heart or respiratory failure, or hypotension [10, 11, 14]. MILA occurs during acute overdose of metformin in the case of suicide attempts, and can induce severe lactic acidosis. However, unlike type A lactic acidosis, where the underlying cause cannot be easily corrected or removed, the prognosis in MILA is relatively good, and patients with blood lactate as high as 40 mmol/L may survive [15].

The aim of the present study was to compare, between metformin users and nonusers, the prognosis of extremely elevated plasma lactate levels and septic shock at arrival to the ER.

## Methods

The electronic medical records (EMR) of all patients admitted to the ER of Assaf Harofeh Medical Center in Israel between 1 January 2011 and 1 June 2013 were reviewed. The study comprised a cohort of patients diagnosed with septic shock and severe lactic acidosis. The study protocol was approved by the Assaf Harofeh Institutional Review Board (IRB).

Study inclusion criteria were age older than 18 years, blood lactate higher than 10 mg/dL and an initial diagnosis of septic shock (ICD-9 codes 995.90–94) in the patients’ files. Septic shock is diagnosed by the ER physician when there is an objective (or assumed) infectious origin/site, together with hemodynamic instability, inflammatory markers and organ dysfunction. Patients with seizures, acute bleeding, acute surgical conditions or trauma were excluded from the study. The selected population was divided into two groups: metformin users (exposed) comprised patients who were actively treated with metformin for at least 3 months just prior to their admission; and metformin nonusers (unexposed) comprised patients who did not meet this criterion.

The clinical standard in the ER is that blood chemistry and blood gases are analyzed for each patient with signs of shock. If high anion gap metabolic acidosis is detected, lactate level is measured. Thus, the EMR data regarding patient analysis on admission could be effectively screened. In addition, patients’ baseline characteristics, including their medical history, presence of diabetes, and blood creatinine levels were recorded. Acute kidney injury (AKI) was determined using risk, injury, failure, loss, end stage (RIFLE) criteria, defined as a twofold increase in serum creatinine, or an estimated glomerular filtration rate (eGFR) (according to the Modification of Diet in Renal Disease (MDRD) study equation) decrease by 50 percent compared to baseline [16]. Preadmission creatinine value was defined as the last value obtained from the hospital’s database or from the managed care organization (outpatient) database. Baseline values were used if they were obtained within the year prior to the relevant admission.

Patient medications were accessed from recordings by ER physicians, based on medical records or anamnesis taken from patients or from those accompanying them. Serum lactate (mmol/L) level was measured using a serum-based immunoassay (Unicel Synchron, Beckman Coulter Inc., Brea, CA, USA) and blood gases were analyzed in the ER using Cobas b 221 (Roche Diagnostics, Basel, Switzerland). Acute physiology and chronic health evaluation (APACHE) II score was calculated for all patients admitted to the ICU, using their values at the first 24 hours.

The primary outcome measure was inhospital mortality. Patients were followed until hospital discharge. Time to event was defined as the time from hospitalization to discharge or death.

### Statistical analysis

Data are expressed as mean ± standard deviation (SD), or median and interquartile range (Q1 to Q3), for variables that did not follow a normal distribution, and as percentages for categorical variables. Normally distributed continuous variables were compared between the metformin user and nonuser groups by means of a two-sided *t* test and with chi-square tests for categorical variables. Since the APACHE II score, creatinine level at admission, and preadmission creatinine levels were not normally distributed, median scores were used for comparisons using the nonparametric Mann–Whitney *U* test. Univariate and multivariate Cox regression analyses are presented as Cox analysis hazard ratios (HR) with confidence intervals (CI). The selection of variables for multivariate models was based on a univariate analysis of each variable. Any variable having a significant univariate test at some arbitrary level was selected as a candidate for the multivariate analysis. We based this on the *p* value cutoff point of 0.25. Survival analyses were performed using the Kaplan–Meier survival curve and the Cox proportional hazard model.

All statistical tests were two-sided, with a value for *p* <0.05 defining significance.

All statistical analyses were performed using SPSS software, version 16.0 (SPSS Inc., Chicago, IL, USA).

## Results

**Fig. 1:**
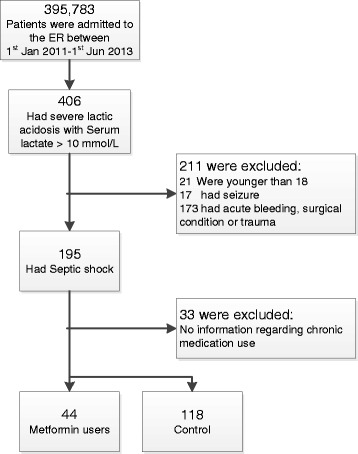
Patient flow

Of 395,783 patients admitted to the ER during the study period, 195 were with septic shock and severe lactic acidosis (blood lactate level >10 mmol/L) (Fig. 1). Thirty-three patients were excluded from analysis due to uncertainty regarding their chronic medication use. Forty-four of the recruited patients were chronically treated with metformin. The 118 patients who were not treated with metformin served as the unexposed group.

Baseline patient characteristics are summarized in Table 1. There were no statistically significant differences between the groups regarding levels of lactate or HCO_3_, or blood pH at admission. However, compared to the control group, metformin users were older 74.0 ± 11.3 vs. 68.4 ± 19.7 years (*p* = 0.03), and had higher cardiovascular risk due to higher incidence of diabetes mellitus, ischemic heart disease and cerebrovascular attacks. Mean preadmission creatinine levels were similar, 1.2 mg/dL vs. 1.1 mg/dL for metformin users and nonusers, respectively, (*p* = 0.36). However, metformin users had higher mean creatinine levels at admission (2.87 vs. 2.14, *p* = 0.02), and higher incidence of AKI (Table 1). Both groups had very high APACHE II score (and thus expected mortality); however, the mean APACHE II score among metformin users was higher, 41.12 ± 8.8 vs. 37.97 ± 6.16, *p* = 0.01. The mean metformin dose was 1.6 ± 0.8 g per day. Suspected origins of infection were confirmed for most of the 44 metformin users: 22 (50 %) had bacteremia, 5 (12 %) had bacteriuria, 3 (7 %) had pneumonia on chest X-ray and 5 (11 %) had skin or wound infection.

**Table 1:** Baseline patient characteristics

	Metformin users (n = 44)	Nonusers (n = 118)	*p* value^*^
Gender (male), n (%)	22 (50 %)	71 (60.17 %)	0.09
Age (mean ± SD)	74.0 ± 11.3	68.4 ± 19.7	0.03
Baseline creatinine	1.2 (0.7–1.3)	1.1 (0.7–1.2)	0.36
Hypertension, n (%)	34 (77.3)	26 (52.5)	<0.001
ACEi, n (%)	18 (40.9)	26 (22.0)	0.29
Beta blockers, n (%)	27 (61.4)	37 (31.4)	0.09
Diabetes, n (%)	44 (100)	23 (19.83)	<0.001
Metformin dose (g)	1.6 (0.8)		
Insulin n (%)	9 (20.5)	13 (11.4)	<0.001
Ischemic heart disease, n (%)	15 (34.1)	358 (29.7)	0.05
Cerebrovascular attack, n (%)	14 (32.6)	23 (19.5)	0.02
Aspirin use, n (%)	20 (45.5)	318 (26.3)	0.65
Malignancy, n (%)	8 (18.2)	21 (17.8)	<0.001
Lactate (median, IQR)	12.7 (11.1–15.0)	12.7 (11.3–16.3)	0.90
Ph (median, IQR)	7.06 (6.9–7.20)	7.10 (6.94–7.20)	0.80
HCO_3_ (mean ± SD)	10.9 ± 5.6	11.5 ± 5.1	0.56
APACHE II score	41.12 ± 8.8	37.97 ± 6.16	0.01
Mortality score	34.7(11.7–55.6)	43.9 (31.5–59.6)	0.19
Creatinine at admission	2.87 (1.5–3.32)	2.14 (1.21–3)	0.02
Acute kidney injury (%)	38 (86.7 %)	65 (56.7 %)	0.02
Renal replacement therapy (%)	(38.6 %) 17	25 (21.2 %)	0.13
Inhospital mortality	25 (556.8 %)	104 (88.1 %)	<0.001
Time to event (hospitalization to death or discharge, days, mean ± SD)	11.67 ± 15.3	4.77 ± 9.79	<0.001

Data presented as mean ± standard deviation (SD) or median and interquartile (IQR) range for parameters not normally distributed or as % for categorical variables

*ACEi* angiotensin-converting enzyme inhibitor, *APACHE II* acute physiology and chronic health evaluation II

^*^ *p* for *t* test or for Mann–Whitney test for parameters not normally distributed or chi^2^ for categorical

The inhospital mortality rate was significantly lower in the metformin-treated group, 56.8 % compared to 88.1 %, despite the more severe risk factors in the former. The Cox analysis HR for inhospital mortality for the metformin group was 0.439, *p* <0.0001.

In a risk factor analysis, age, diabetes and metformin use were significantly associated with the risk for inhospital mortality, with HRs of 1.05 (1.01–1.08), *p* = 0.05; 0.5 (0.34–0.74); *p* = 0.01; and 0.42 (0.27–0.66), respectively (Table 2). For the high range of lactate levels of the present cohort (all greater than 10 mmol/L), lactate level had no prognostic value for the entire cohort, or for metformin users or for nonusers separately (HR = 1.06 (0.99–1.1), *p* = 0.284, HR = 1.04 (0.96–1.16), *p* = 0.129 and HR = 1.05 (0.99–1.11), *p* = 0.091, respectively (Table 2).

**Table 2:** Hazard ratios and confidence intervals for inhospital mortality

Variable	All patients	Metformin users	Metformin nonusers
	N = 162	n = 44	n = 118
Age (1 year↑)	1.05 (1.02–1.08)^P^	0.99 (0.95–1.02)	1.05^P^ (1.0–1.08)
Gender (male)	0.83 (0.58–1.18)	0.67 (0.30–1.14)	0.88 (0.59–1.32)
Diabetes	0.50 (0.34–0.74)^P^	---	0.82 (0.48–1.40)
Hypertension	0.79 (0.55–1.14)	0.47 (0.19–1.14)	1.12 (0.74–1.65)
IHD	1.22 (0.83–1.78)	0.88 (0.37–2.06)	1.55^P^ (1.01–2.4)
Cerebrovascular attack	1.09 (0.72–1.65)	1.57 (0.69–3.50)	1.15 (1.01–2.40)
Lactate (1 mg/dL↑)	1.06 (0.99–1.10)	1.04 (0.96–1.16)	1.05 (0.99–1.11)
pH	0.91 (0.37–2.26)	1.02 (0.16–6.45)	1.05 (0.99–1.11)
Bicarbonate (1 meq/L↑)	0.97 (0.93–1.01)	0.99 (0.92–1.06)	0.97 (0.93–1.01)
APACHE II (1↑)	1.03 (0.99–1.07)	1.10 (1.01–1.20)^P^	0.99 (0.95–1.04)
Acute kidney injury	0.93 (0.62–1.40)	0.80 (2.37–2.68)	0.91 (0.54–1.50)
Creatinine (1 mg/dL↑)	0.92 (0.82–1.00)	0.91 (0.75–1.11)	1.08 (0.92–1.29)
Anion gap (1↑)	0.99 (0.97–1.01)	0.98 (0.94–1.02)	1.01 (0.98–1.04)
HD	0.61 (0.40–0.95)^P^	0.46 (0.19–1.11)	0.91 (0.54–1.50)
Temperature (1 °C)	0.94 (0.63–1.40)	0.89 (0.55–1.34)	0.98 (0.67–1.45)
Metformin	0.42 (0.27–0.66)^P^	---	---
Metformin adj^*^	0.21 (0.05–0.94)^P^	---	---

Univariate analyses are presented for the investigated variables. A multivariate analysis is presented for metformin use

*IHD* ischemic heart disease, *HD* underwent hemodialysis, *APACHE II* acute physiology and chronic health evaluation II.

^P^Refers to *p* <0.05

^*^Adjusted for age, gender, diabetes, hypertension, ischemic heart disease, cerebrovascular disease, malignancy, levels of creatinine and lactate at admission, and APACHE II score

**Fig. 2:**
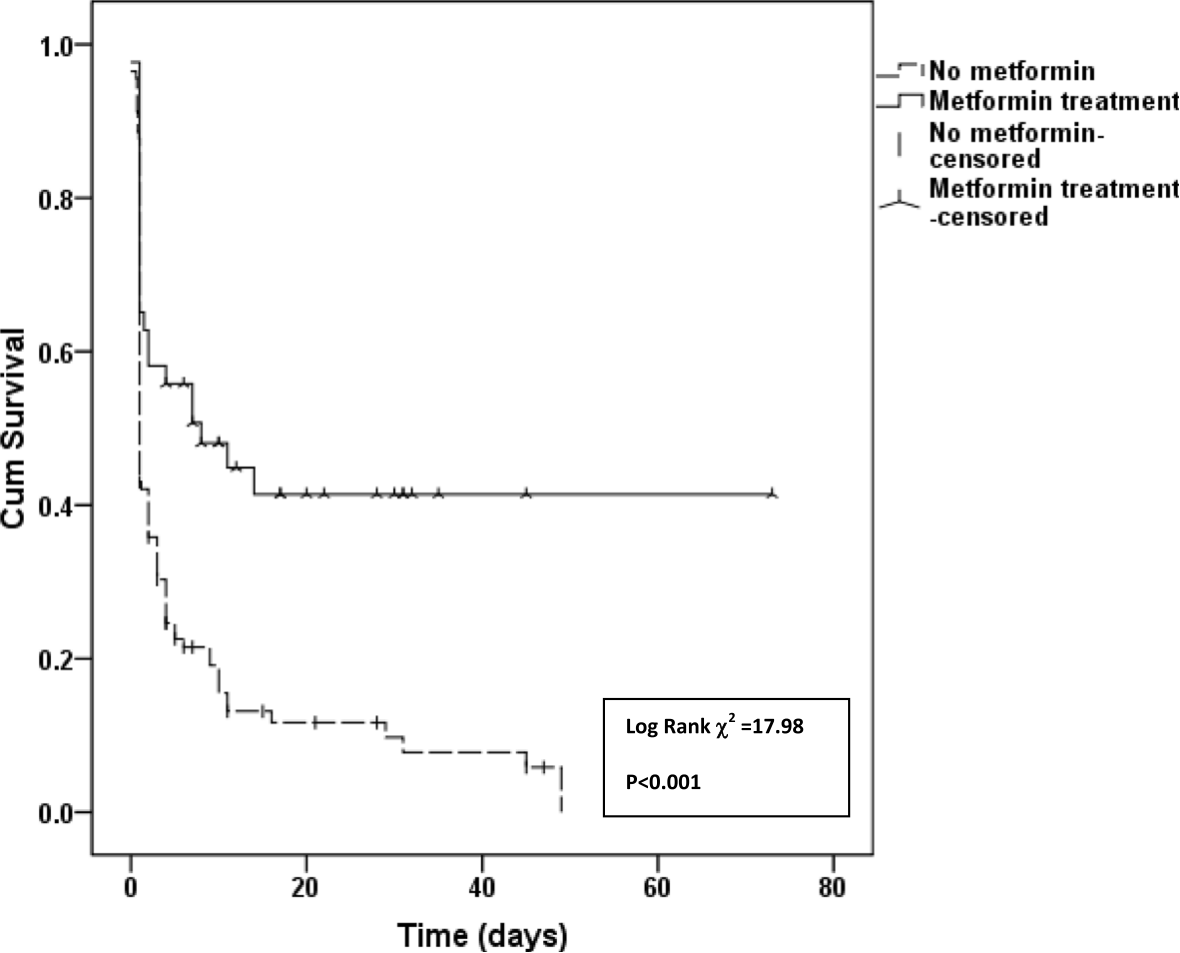
Kaplan–Meier survival curve

In a multivariate analysis, that adjusted for age, gender, diabetes, hypertension, ischemic heart disease, cerebrovascular disease, malignancy, levels of creatinine and lactate at admission, and APACHE II score, the HR for inhospital mortality among metformin users was 0.207 (CI 0.046–0.939), *p* = 0.041. Kaplan–Meier curves showed greater cumulative survival for the metformin group during hospitalization (Fig. 2).

Survivors and nonsurvivors among metformin users were further compared. Among metformin users, survivors showed higher incidence of acute renal failure and a greater frequency of hemodialysis, yet lower APACHE scores, than did nonsurvivors (Table 3).

**Table 3:** A comparison, among metformin users, of clinical characteristics between survivors and nonsurvivors

Variable	Survivors	Nonsurvivors	
	N = 19	n = 25	*p*
pH (mean ± SD)	7.06 ± 0.29	7.05 ± 0.16	0.93
Bicarbonate (mEq/L) (mean ± SD)	11.6 ± 6.4	10.4 ± 5.2	0.52
APACHE II (mean ± SD)	16.0 ± 6.1	28.8 ± 8.2	0.006
Morscore^*^ (median, range)	22.0 (9.7–34.5)	59.6 (37.7–78.9)	0.01
Creatinine^*^ (md/dL) (median, range)	1.65 (1.29–5.08)	1.95 (1.50–2.88)	0.73
Pread cre^*^ (mg/dL) (median, range)	0.85 (0.66–1.70)	0.87 (0.70–1.15)	0.83
Anion gap (mean ± SD)	30.4 ± 10.0	28.1 ± 8.6	0.46
HD^**^ (%)	52.6	28.0	0.028
Body temperature (°C) (mean ± SD)	36.2 ± 1.5	36.0 ± 1.0	0.67

Data are represented as means ± standard deviations (SD), medians and ranges, or percentages

*APACHE II* acute physiology and chronic health evaluation II, *Morscore* mortality score, based on APACHE II score, *Pread cre* preadmission creatinine, *HD* hemodialysis

*p* for *t* test, ^*^Mann–Whitney or ^**^chi-square

## Discussion

In a cohort of patients admitted to the ER with severe lactic acidosis and sepsis, the rate of inhospital mortality was lower for those who were actively treated with metformin than for those who were not, 56.8 % vs. 88.1 %, *p* <0.0001. The unexpected relatively high survival rate of metformin users distinguishes them as patients who may benefit from intensive treatment.

Lactate was first suggested as a clinical prognostic tool by Broder and Weil in 1964 when they observed that a level of >4 mmol/L was associated with poor outcomes in patients with undifferentiated shock [17, 18]. Since then, much has been published on the etiology and treatment of elevated lactate levels in a variety of patient populations [17, 18]. The role of blood lactate as a marker of tissue hypoperfusion has demonstrated prognostic value [3, 4, 19]. Moreover, hyperlactatemia has been suggested as an obligatory diagnostic criterion for septic shock [19]. The current study examined patients with septic shock and extremely severe lactic acidosis, represented by blood lactate higher than 10 mmol/L at hospital admission. The very high mortality rate observed further supports the prognostic value of high lactate levels in risk assessment of septic patients. However, the prognostic value of lactic acidosis in metformin users was different, as reflected by better survival. Their lower mortality rate is particularly remarkable in light of the higher risk profile of the metformin users: older mean age, higher incidence rates of diabetes, cardiovascular diseases and AKI.

Septic shock is clearly a condition of hypoperfusion and hypoxemia that predisposes to lactate generation. However, sepsis may also alter metformin metabolism. Metformin is eliminated rapidly and actively by the kidney and any decrease in renal perfusion and/or acute tubular injury, as happens during AKI, may lead to metformin accumulation [20]. Though metformin users in the current study were treated with standard doses, not exceeding 2550 mg/day, 86.7 % of them had AKI, which may predispose to metformin accumulation. While the prognostic value of sepsis-induced lactic acidosis is severe, the prognosis of pure metformin intoxication is significantly better (patients with blood lactate as high as 40 mg/dL may survive).

In addition to metformin accumulation and amplification of hypoxic-induced lactate generation, metformin has pleiotropic effects that may be beneficial during critical illness. Preclinical and clinical studies showed that metformin has anti-inflammatory [21, 22] and antithrombotic [23] effects. Metformin induces activation of AMP-activated protein kinase (AMPK) [19, 20], an enzyme that is normally induced by cellular stress and hypoxia/anoxia [23, 24]. Once activated, AMPK regulates cellular energy status; it switches on the ATP-generating pathways and switches off the ATP-consuming pathways [7]. This switch improves cellular function under stress conditions, such as improved cardiovascular function during stress [25, 26].

The beneficial effect versus risk of metformin administration in patients suffering from chronic kidney disease (CKD) is a debatable issue. In the current study, population preadmission creatinine levels of patients under metformin treatment were within FDA guidelines (27), which state that creatinine levels higher than 1.5 mg/dL in males and 1.4 mg/dL in females, is a contraindication to the use of metformin. Preadmission creatinine was not a risk factor for lactate accumulation; it was acute rather than chronic renal failure that was associated with lactic acidosis. However, patients with CKD are at high risk for AKI leading to metformin accumulation and lactate generation. Considering the beneficial effects of metformin for type II diabetes, the findings of this study may support metformin administration to patients with mild and stable CKD, but the risk of AKI should always be considered.

The current study has several limitations. For one, lack of measurement of circulating metformin levels precludes verifying an association between severe lactic acidosis and metformin accumulation. An additional limitation relates to the higher risk for lactic acidosis in metformin users than in nonusers. While this could be expected to result in selection bias, as metformin users may be more likely to have lactate measurements, the higher mean APACHE score in the metformin group does not support such possibility.

Lastly, to achieve a “clear-cut” very high-risk population, only patients with extremely elevated blood lactate (>10 mmol/L) were included in the study. The upshot is that conclusions for the effect of metformin in mild lactic acidosis cannot be drawn.

## Conclusions

Though high lactate concentration in septic shock patients indicates poor prognosis, prognosis was found to be less severe among metformin users. This finding may help clinicians in deciding treatment for these patients, who could otherwise be considered too ill for real treatment benefit.

## Key messages

- High lactate concentration in septic shock patients treated with metformin appears to differ from that presenting in patients who do not use metformin.
- The prognosis of metformin users suffering from septic shock with severe lactic acidosis is significantly better than that of patients who are not metformin users.

## Abbreviations

AKI: Acute kidney injury
AMPK: AMP-activated protein kinase
APACHE: Acute physiology and chronic health evaluation
CKD: chronic kidney disease
EMR: Electronic medical records
ER: Emergency room
ICU: Intensive care unit
MALA: Metformin-associated lactic acidosis
MILA: Metformin-induced lactic acidosis
